# Encysted Peritoneal Tuberculosis Masquerading as Ovarian Tumour: A Case Report

**DOI:** 10.7759/cureus.39956

**Published:** 2023-06-04

**Authors:** Farah P Jiandani, Savita Somalwar, Anuja Bhalerao, Sheela Jain, Shraddha Rao

**Affiliations:** 1 Department of Obstetrics and Gynaecology, NKP Salve Institute of Medical Sciences and Research Centre, Nagpur, IND

**Keywords:** ca-125, laparotomy, extrapulmonary tuberculosis, ovarian tumour, peritoneal tuberculosis

## Abstract

Peritoneal tuberculosis (TB) is one of the types of extrapulmonary TB that predominantly involves the omentum, liver, intestinal tract, spleen, or female genital tract. It can occasionally result in gynecological-related oncology diagnoses such as advanced ovarian cancer due to its non-specific signs and symptoms, making it very difficult to detect. This report presents a case of a 22-year-old female who presented with the chief complaints of pain and distension of the abdomen for one month with dysuria. Ultrasonography and magnetic resonance imaging was performed that reported a large uni-loculated cystic pelvic abdominal lesion likely to be of ovarian origin and suggestive of neoplastic etiology with bilateral hydroureteronephrosis. To confirm the diagnosis, an exploratory laparotomy was performed which revealed extrapulmonary abdominal TB, and was registered for Directly Observed Treatment Shortcourse (DOTS) following which anti-tubercular drugs were given. In conclusion, this case report highlighted the masquerading behavior of encysted peritoneal TB as an ovarian tumor, and the fact that it should, therefore, should be considered in the differential diagnosis in regions where TB remains endemic, such as in developing countries. Hence, an appropriate diagnosis can prevent the need for unnecessary surgical operations and adequate therapy can save the patient's life.

## Introduction

Peritoneal tuberculosis (TB), which is an extrapulmonary TB, mainly involves the intestinal tract, omentum, liver, spleen, or female genital tract [1]. It affects 1-2% of patients with pulmonary TB [2]. Due to its non-specific signs and symptoms, it is extremely challenging to detect and occasionally causes gynecological-related oncology diagnoses such as advanced ovarian carcinoma (OC) [2]. Globally, out of 10 million cases, India alone carries the highest TB burden accounting for 2.8 million cases [3]. Abdominal TB, which is less frequent than pulmonary TB, can lead to severe morbidity and mortality and is usually detected much later than pulmonary TB [3,4]. It mainly represents four forms that include visceral TB involving the solid organs, lymph nodal TB, gastrointestinal TB, and peritoneal TB [3,4].

Common findings of peritoneal TB include ascites, mesenteric adhesions, lymphadenopathy, septation in the ascites, and omental involvement that considerably overlap with the clinical presentation of primary peritoneal carcinoma (PPC) or advanced OC, which consists of a heterogeneous mass in addition to the above-mentioned findings leading to a misdiagnosis of advanced malignancy [5]. Serum cancer antigen (CA) 125 can be elevated in both peritoneal TB and OC [3].

OC is the seventh most common cancer in women worldwide in terms of mortality related to cancer [6]. The most typical sign of OC is an adnexal mass on pelvic examination or imaging and its non-specific symptoms consist of unspecific pelvic or abdominal pain, abdominal distension, early satiety, and urinary symptoms [6]. Additionally, imaging studies consisting of computed tomography (CT) scans and ultrasonography (USG) should be considered for the purpose of diagnosis [1]. Therefore, in the differential diagnosis of OC, abdominal TB with ascites must be taken into account because it can mimic widespread OC [7]. Furthermore, being able to rule out malignancy as soon as possible is much beneficial in terms of treatment and prognosis.

## Case presentation

A 22-year-old female, nulligravida, presented with the chief complaints of pain and distension of the abdomen for one month with dysuria. The patient reported a history of weight loss for two months with a normal appetite. There was no significant past medical or surgical history except for the need for a blood transfusion in 2014 due to anemia. There was no significant family history of TB and allergies; however, the patient had a secondary contact three months prior to the date of the emergence of chief complaints.

On examination, the patient had a thin build with a weight of 40 kg and a BMI of 16.2 kg/m^2^. She had pallor and tachycardia (pulse 120/minute) with bilateral pedal edema and normal blood pressure. The abdomen was distended up to the xiphisternum and there was a cystic mass of around 24 weeks per uterine size, having a smooth surface with restricted mobility due to Grade 1 tenderness. Additionally, there was no evidence of any lymphadenopathy or any other systemic involvement.

**Figure 1:**
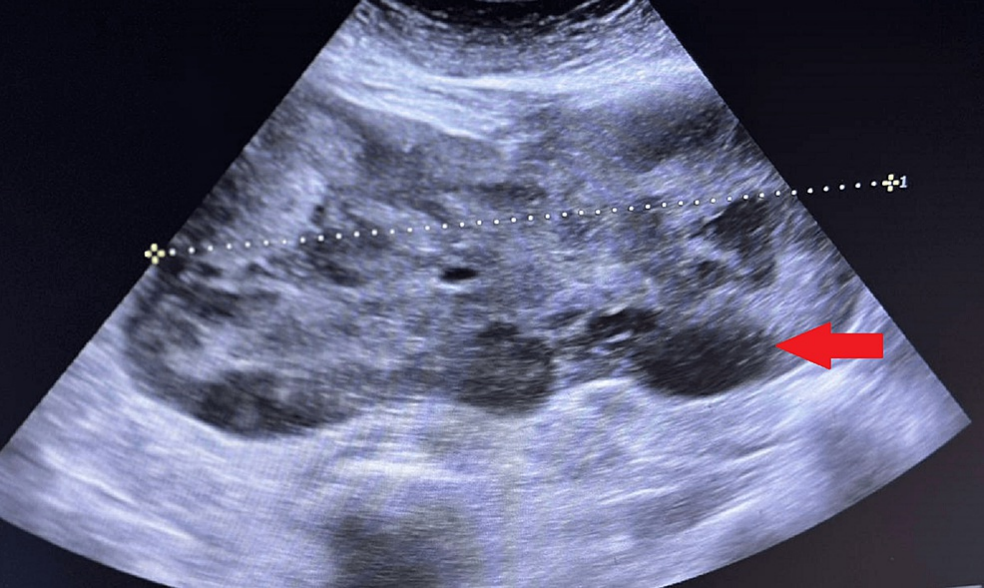
Ultrasonography demonstrating anechoic cystic mass

The USG of the abdomen and pelvis revealed 29x25x25 cm thick-walled (3.3 mm) well-defined anechoic cystic mass lesions with a volume of 9062cc with thin septation. The probable diagnosis made on USG was a well-defined anechoic cystic lesion noted in the right adnexa, likely of ovarian origin, as depicted in Figure 1.

**Figure 2:**
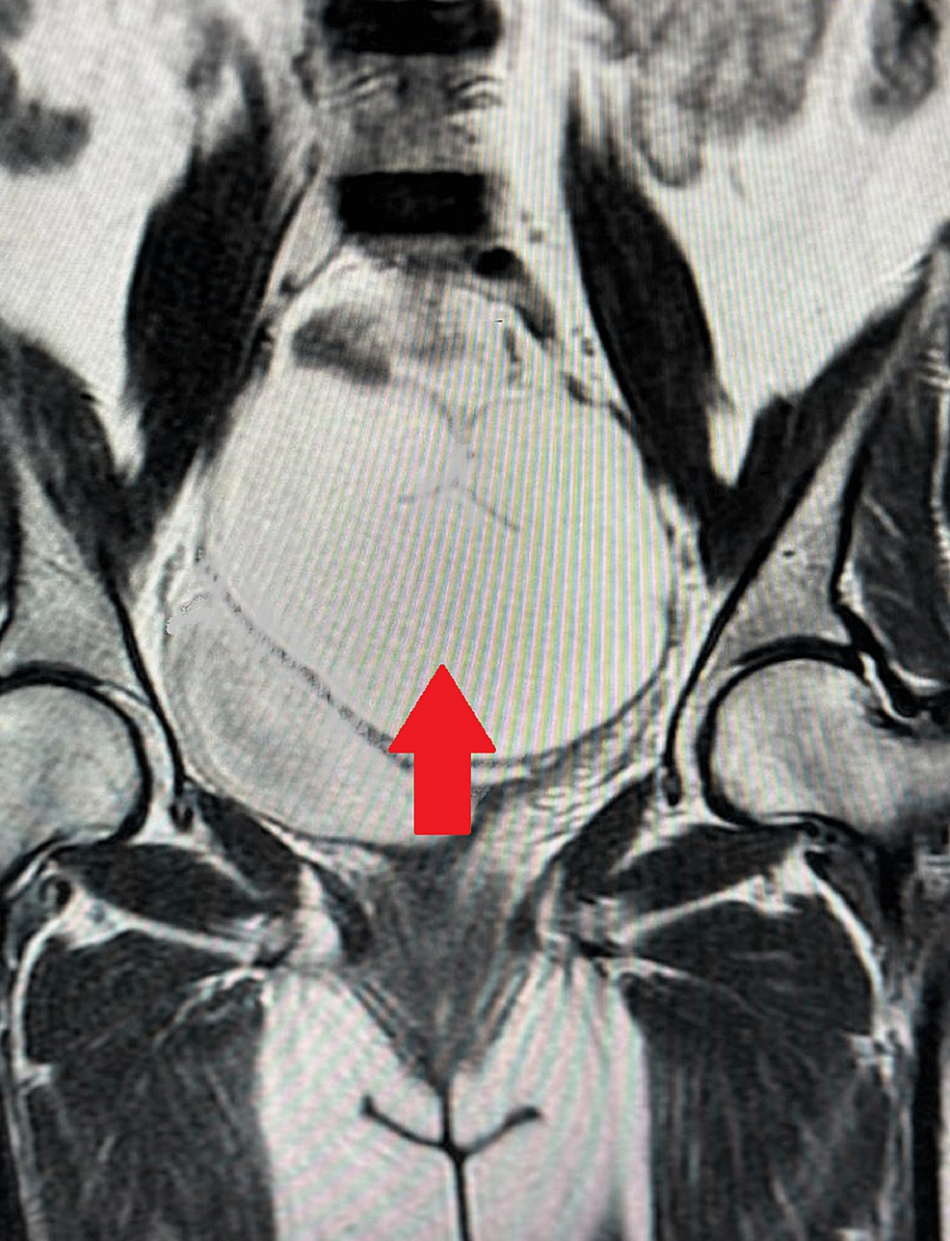
Magnetic resonance image illustrating large uni-loculated cystic pelvic abdominal lesion

For a definitive diagnosis, magnetic resonance imaging (MRI) was performed. The MRI report was suggestive of a large uni-loculated cystic pelvic abdominal lesion epicentered in the pelvis, arising from the right adnexa, likely of ovarian origin, with bilateral hydroureteronephrosis as shown in Figure 2.

**Figure 3:**
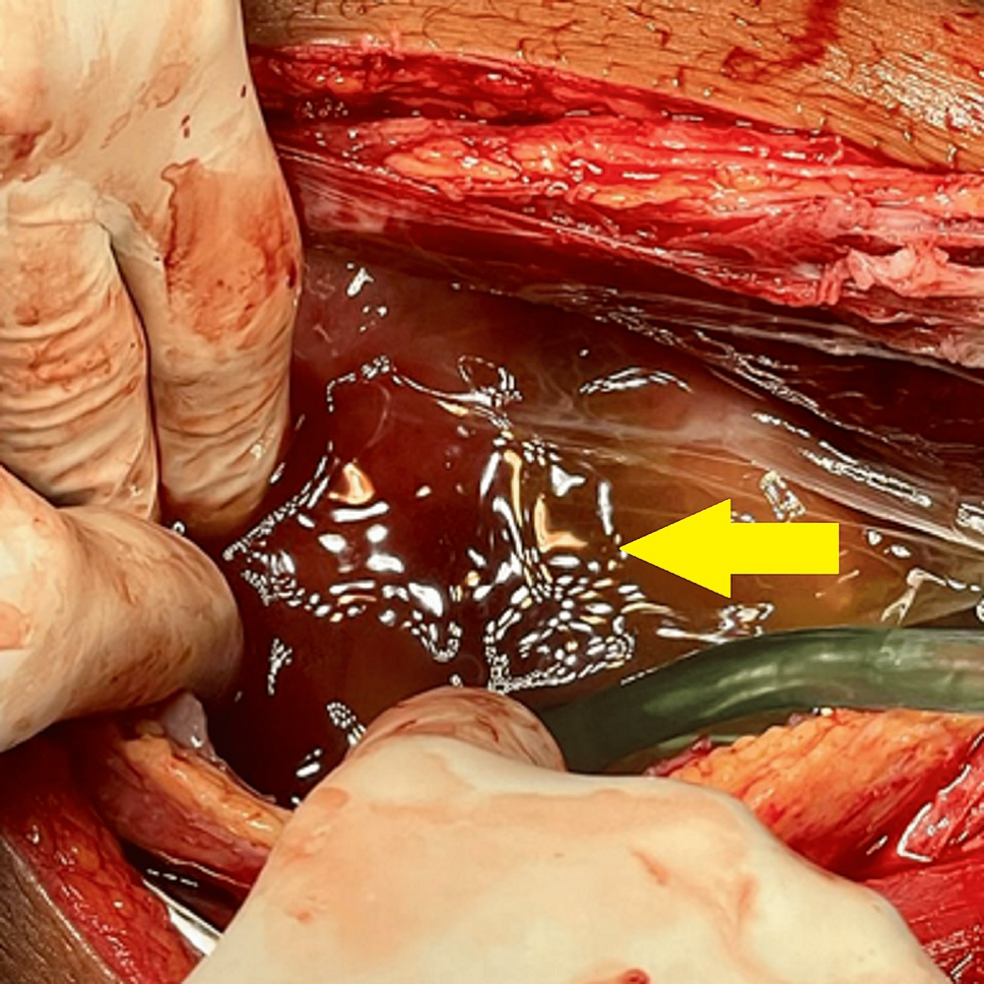
Exploratory laparotomy showing ascites (yellow arrow)

The biochemical evaluation revealed reduced hemoglobin of 8.2 g/dl (normal reference range: 12-16 g/dl) and raised total leukocyte count of 10,950/mm^3^ (normal reference range: 4,500-10,500/mm^3^ for adults). The CA-125 was 523 U/ml (reference range: 0-35 U/ml), serum lactate dehydrogenase (LDH) was slightly raised at 285 U/L (reference range of 140-280 U/L), and beta-human chorionic gonadotropin (HCG) was <2 mIU/mL (reference range: 0-5 mIU/mL). These clinical, laboratory, and radiological findings favored the diagnosis of ovarian malignancy. Furthermore, to confirm the diagnosis, exploratory laparotomy was performed as shown in Figure 3.

**Figure 4:**
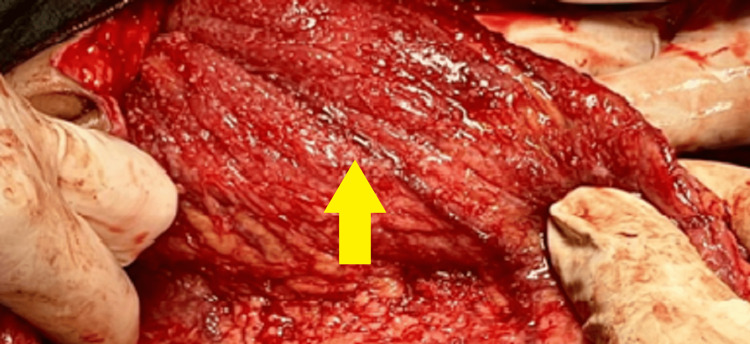
Multiple tubercles present on the posterior surface of the omentum

On exploratory laparotomy, intraoperative findings demonstrated that the peritoneum was thickened, and densely adhered to the omentum, and assistance was obtained from the surgeon to separate the omentum. Multiple tubercles were present on the posterior surface of the omentum, as illustrated in Figure 4. A right-sided ovarian mass was seen along with a thick-walled cystic mass of 7x6x5 cm that was present centrally. Approximately 2000 cc of peritoneal fluid was drained and post-peritoneal wash and closure were done and hemostasis was achieved.

The postoperative course was uneventful. The Mantoux test was performed to confirm the diagnosis of TB and reported negative results. The histopathological examination of the omental biopsy and peritoneal fluid showed signs of granulomatous inflammation suggesting TB. The chest x-ray demonstrated no effusions or infiltrations. The patient was diagnosed with extrapulmonary abdominal TB and was registered for Directly Observed Treatment Shortcourse (DOTS) following which anti-tubercular drugs were given. Monthly sonography was done as a follow-up to evaluate the condition of the patient in which the patient had a good clinical response after six months of treatment. CA 125 was used to monitor the treatment. Six-month follow-up values of CA 125 after the treatment were found to be 38 U/mL after two months, 12 U/mL after four months, and 8 U/mL after six months.

## Discussion

This report highlights a rare case of encysted peritoneal TB masquerading as an ovarian tumor, as TB can affect any organ of the body. Peritoneal TB is quite challenging to differentiate from malignant tumors of the abdomen as there can be similarities in symptoms which consists of abdominal pain, fever, abdominal swelling, weight loss, and high CA 125 levels [8]. The hematogenous transfer of *Mycobacterium tuberculosis* from a pulmonary infection to the abdominal cavity may be the cause of peritoneal TB. The primary lung focus typically heals completely with no clinical or radiological signs [1,4].

In the present case, the biochemical findings reported reduced hemoglobin levels, raised total leukocyte count, increased CA 125, and raised serum LDH; beta-HCG was within normal limits. In PCC cases, CA 125 levels are usually higher but, it should be noted that in patients who have peritoneal TB, CA 125 can elevate to about 10-fold of the normal value [8]. According to a prior study on peritoneal TB, abdominal pain seen in 70% of patients and distension seen in 65% of patients are the symptoms and signs, respectively, that are mostly seen during physical examination. According to the laboratory results of a study by Oge et al. that reported peritoneal TB mimicking OC, the average level was 289±186.2 IU/mL, in which 75% had levels greater than 100 IU/mL and 80% of patients had CA 125 levels greater than 35 IU/mL [9]. In a retrospective study, the mean CA 125 was 666.9 U/ml in patients diagnosed with peritoneal TB [10]. As CA 125 levels are also raised in malignancies [6,8], these raised levels of CA 125 can lead to obstruction in differentiating malignancy from TB.

Additionally, in the present case, the peritoneum was thickened, and densely adhered to the omentum, and multiple tubercles were present on the posterior surface of the omentum. Similarly, in previous studies by Bulut et al. and Laitaifeh et al. demonstrating a challenging diagnosis of peritoneal TB and disseminated peritoneal tuberculosis mimicking advanced OC, the imaging examination concluded that ascites were the main findings followed by omental and peritoneal thickening [8,11]. Moreover, adnexal and fallopian masses, ascites, nodular irregularities in the peritoneal surface, and septated and multiloculated ovarian cysts are all radiographic findings of the abdomen that are extremely similar in both peritoneal TB and OC [8]. In this case, an exploratory laparotomy was done to confirm the diagnosis as it is considered a safe and reliable method of diagnosis, particularly when ascites are present [12,13]. Furthermore, early diagnostic workup with an USG-guided laparoscopic biopsy has been found to confirm the diagnosis accurately [3].

Therefore, peritoneal TB must always be taken into account while making a differential diagnosis of OC, particularly in economically poor or undeveloped nations. Additionally, medical treatment consisting of anti-tubercular drugs for abdominal TB is usually effective. The usual course of treatment involves a three-drug regimen of isoniazid, rifampin, and pyrazinamide for two months, followed by four months of isoniazid and rifampin. If drug resistance is suspected, due to prior therapy with isoniazid alone, a fourth drug, typically ethambutol, or a fifth, such as streptomycin, may be added to the initial two-month intensive phase [14]. Hence, early detection, evaluation, and treatment can avoid the need for surgical intervention.

## Conclusions

This case report highlights the masquerading behavior of encysted peritoneal TB as an ovarian tumor and it should, therefore, should be considered in the differential diagnosis of OC, especially in regions where TB remains endemic such as in developing countries. However, the diagnosis is rarely easy for clinicians. Additionally, as the treatment for OC and peritoneal TB differs, it is important to correctly diagnose lower abdominal abnormalities as described in this case. A correct diagnosis can prevent the need for unnecessary surgical operations using pre-operative minimally invasive techniques, and appropriate therapy and follow-up can save the patient's life.
